# Determinants of intramyocellular lipid accumulation in early childhood

**DOI:** 10.1038/s41366-019-0435-8

**Published:** 2019-08-28

**Authors:** Navin Michael, Varsha Gupta, Suresh Anand Sadananthan, Aparna Sampathkumar, Li Chen, Hong Pan, Mya Thway Tint, Kuan Jin Lee, See Ling Loy, Izzuddin M. Aris, Lynette Pei-Chi Shek, Fabian Kok Peng Yap, Keith M. Godfrey, Melvin K.-S. Leow, Yung Seng Lee, Michael S. Kramer, Christiani Jeyakumar Henry, Marielle Valerie Fortier, Yap Seng Chong, Peter D. Gluckman, Neerja Karnani, S. Sendhil Velan

**Affiliations:** 1 Singapore Institute for Clinical Sciences, Agency for Science Technology and Research, Singapore, Singapore; 2 Department of Obstetrics and Gynaecology, Yong Loo Lin School of Medicine, National University of Singapore, Singapore, Singapore; 3 Singapore Bioimaging Consortium, Agency for Science Technology and Research, Singapore, Singapore; 4 Department of Reproductive Medicine, KK Women’s and Children’s Hospital, Singapore, Singapore; 5 Duke-NUS Medical School, Singapore, Singapore; 6 Department of Paediatrics, Yong Loo Lin School of Medicine, National University of Singapore, Singapore, Singapore; 7 Department of Paediatrics, KK Women’s and Children’s Hospital, Singapore, Singapore; 8 Lee Kong Chian School of Medicine, Nanyang Technological University, Singapore, Singapore; 9 Department of Obstetrics and Gynaecology, KK Women’s and Children’s Hospital, Singapore, Singapore; 10 MRC Lifecourse Epidemiology Unit & NIHR Southampton Biomedical Research Centre, University of Southampton & University Hospital Southampton NHS Foundation Trust, Southampton, UK; 11 Clinical Nutrition Research Centre, Singapore Institute for Clinical Sciences, Agency for Science Technology and Research and National University Health System, Singapore, Singapore; 12 Department of Endocrinology, Tan Tock Seng Hospital, Singapore, Singapore; 13 LKC School of Medicine, Nanyang Technological University Singapore, Singapore, Singapore; 14 Department of Biochemistry, Yong Loo Lin School of Medicine, National University of Singapore (NUS), Singapore, Singapore; 15 Departments of Pediatrics and of Epidemiology, Biostatistics and Occupational Health, Faculty of Medicine, McGill University, Montreal, QC Canada; 16 Department of Biochemistry, Yong Loo Lin School of Medicine, National University of Singapore, Singapore, Singapore; 17 Department of Diagnostic and Interventional Imaging, KK Women’s and Children’s Hospital, Singapore, Singapore; 18 Liggins Institute, University of Auckland, Auckland, New Zealand

**Keywords:** Obesity, Translational research

## Abstract

**Background/Objectives:**

Accumulation of lipid droplets inside skeletal muscle fibers (intramyocellular lipids or IMCL) with increasing obesity has been linked to skeletal muscle insulin resistance and risk of type 2 diabetes in both adults and prepubertal children. We aimed to evaluate the associations of race, genotype, prenatal factors, and postnatal factors with IMCL in early childhood.

**Subjects/Methods:**

This study was a secondary analysis performed on the GUSTO birth cohort. Soleus muscle IMCL of 392 children at 4.5 years of age was measured by magnetic resonance spectroscopy, of which usable imaging data were obtained from 277 children (137 Chinese, 87 Malays, and 53 Indians). Metabolic assessments (fasting glucose, insulin, and HOMA-IR) were performed at age 6.

**Results:**

The mean IMCL level at 4.5 years was 0.481 ± 0.279% of water resonance (mean ± sd). Corroborating with results from adults, Indian children had the highest IMCL levels compared with Malay and Chinese children. Among the prenatal factors, the rate of gestational weight gain (GWG rate) was associated with offspring IMCL (*B* = 0.396 (0.069, 0.724); *p* = 0.018). Both race and GWG rate continued to be associated with offspring IMCL even after accounting for current offspring BMI. Postnatally, IMCL was associated with shorter breastfeeding duration (*B* = 0.065 (0.001, 0.128); *p* = 0.045) and conditional relative weight gain between ages 2 and 3 (*B* = 0.052 (0.012, 0.093); *p* = 0.012). The associations with postnatal factors were attenuated after adjusting for current offspring BMI. IMCL was positively associated with offspring BMI (*B* = 0.028 (0.012, 0.044); *p* = 0.001). IMCL levels were not associated with fasting glucose, fasting insulin, and HOMA-IR at age 6.

**Conclusion:**

This study provides evidence that IMCL accumulation occurs in early childhood and that developmental factors and race are associated with it. We also show that early childhood IMCL accumulation is well tolerated, suggesting that the adverse associations between IMCL and insulin resistance may emerge at older ages.

## Introduction

Excessive cytosolic accumulation of lipid droplets within myocytes, referred to as intramyocellular lipids (IMCL), has been linked to insulin resistance (IR) and increased risk of type 2 diabetes mellitus [1–4]. The question of whether IMCL is causally linked to IR has been a matter of considerable debate. IMCL’s association with IR is quite complex and is influenced by muscle fiber type distribution (IMCL levels are higher in insulin sensitive oxidative fibers than glycolytic fibers [5]), exercise capacity (IMCL is positively associated with insulin sensitivity in individuals with high exercise capacity and negatively in individuals with low exercise capacity [6]), sex (women have higher IMCL despite being more insulin sensitive than men [7]), and subcellular localization (subsarcolemmal IMCL is more strongly linked to IR than intermyofibrillar IMCL [8]). While studies in adolescents and preadolescents have indicated IMCL’s link to childhood obesity [9], adiponectin levels [4], IR, and type 2 diabetes [10], it is not clear if IMCL in early childhood is well tolerated or if it is linked to metabolic alterations. The pathophysiological origins of IMCL accumulation in early childhood and the role of early developmental factors have also not been well studied. Among adults, there are marked racial differences in IMCL levels. South-Asians are marked by elevated IMCL levels when compared with Chinese, Malays and Caucasians, even at lower levels of adiposity [11, 12]. The prevalence of type 2 diabetes is also higher in South Asians at younger ages and lower levels of adiposity, when compared with other racial groups [13]. It is not known if the racial differences in IMCL are apparent in early childhood.

In this study we report the first measurements of IMCL using magnetic resonance spectroscopy (MRS) in early childhood. The current work constitutes a secondary analysis performed on the Growing Up in Singapore Towards healthy Outcomes (GUSTO) birth cohort. GUSTO is deeply phenotyped cohort comprising of Chinese, Malay, and Indian (South Asian) participants, which allowed us to examine the hypothesis that variability in IMCL accumulation is associated with exposure to markers of suboptimal in utero environment, such as maternal adiposity (BMI), gestational weight gain (GWG), and maternal glycaemia, and postnatal factors like breastfeeding duration and rapid postnatal weight gain. We assessed the associations of race and genetic variation on early childhood IMCL levels. We also evaluated the association of early childhood IMCL with metabolic outcomes.

## Methods

### Study population

The GUSTO birth cohort was set up to investigate the developmental origins of health and disease [14]. This study was registered at www.clinicaltrials.gov as NCT01174875. The cohort included 1247 pregnant women in their first trimester and of ≥18 years of age, recruited from the National University Hospital (NUH) and KK Hospital (KKH). Excluding the dropouts, 1191 babies were born. This study was approved by the Domain-Specific Review Board of NUH and the Centralized Institutional Review Board of KKH. Magnetic resonance imaging was performed at the 4.5-year time-point and for this 981 parents were approached. Consent for MR imaging was obtained for 503 children, out of which 392 children completed the MRI visit. After quality control, imaging data from 277 children remained for downstream analysis, including 128 boys and 149 girls, and a racial distribution of 137 Chinese, 87 Malays, and 53 Indian children (Table 1).

**Table 1:** Demographic and clinical characteristics of mother and offspring in the three racial groups (mean (sd) for continuous variables and percentages for categorical variables)

Characteristics	Chinese (C)	Malays (M)	Indians (I)	*P*-value^a^	Post Hoc^b^
*N*	137	87	53		
Maternal age (years)	31.9 (5.1)	28.4 (5.5)	30.72 (5.1)	**<0.001**	C > M, I > M
Maternal BMI (kg/m^2^)	22.6 (3.6)	26.4 (5.9)	26.0 (5.4)	**<0.001**	M > C, I > C
Maternal education <12 years	40.0%	73.6%	41.2%	**<0.001**	C↓, M↑
Maternal fasting glucose (mmol/l)	4.3 (0.4)	4.3 (0.6)	4.6 (0.6)	**<0.001**	I > C, I > M
Maternal 2 h glucose (mmol/l)	6.6 (1.3)	5.9 (1.2)	6.6 (1.7)	**<0.001**	C > M, I > M
GWG rate (kg/week)	0.468 (0.094)	0.466 (0.122)	0.443 (0.120)	0.395	
Excessive GWG rate (IOM 2009)	39.3%	61.1%	44.7%	**0.014**	C↓, M↑
Primiparity	37.5%	41.4%	41.5%	0.800	
Gestational age (days)	270.9 (10.3)	266.8 (14.2)	270.49 (12.7)	**0.039**	C > M
Male sex	47.4%	48.3%	39.6%	0.560	
Birth weight (g)	3083 (412)	3021 (553)	3003 (531)	0.491	
Breastfeeding duration <3 months	39.4%	62.7%	52.2%	**0.004**	C↓, M↑
Weight, 4.5 years (kg)	17.3 (2.6)	17.5 (3.0)	18.3 (4.4)	0.127	
Height, 4.5 years (cm)	105.5 (4.3)	104.2 (3.6)	107.5 (4.3)	**<0.001**	I > C, I > M
BMI, 4.5 years (kg/m^2^)	15.5 (1.5)	16.0 (1.9)	15.71 (2.8)	0.071	
Sum of skinfolds, 4.5 years (mm)	29.8 (8.6)	32.9 (12.8)	34.3(17.8)	**0.039**	

Bold values indicate statistical significance *P* < 0.05

^a^*P*-value column indicates significance of ANOVA test for continuous variables and χ^2^ test for categorical variables

^b^Post Hoc column indicates significant pairwise differences in the mean values after multiple comparisons with Bonferroni correction. For categorical variables, the post hoc columns indicates the columns with significantly elevated/reduced values from a column proportion test

### Exposures

#### Maternal and intrauterine exposures

Information about prepregnancy weight, parity, socioeconomic status, and demographics were collected through questionnaires administered upon study enrollment. Maternal height was recorded during the second trimester (26th–28th weeks). During the same visit, maternal glucose levels (fasting and 2-h post glucose load) were measured during an oral glucose tolerance test (75 grams glucose load after an overnight fast (8–14 h)). Serial measurements of maternal weight throughout pregnancy were obtained from the clinical records. Maternal BMI calculated from the weight recorded at the first clinical visit, was used for subsequent statistical analyses which required an adjustment for maternal adiposity. Linear mixed effects model with the best linear unbiased predictor was performed to estimate linear trajectory of GWG per week between 15 to 35 weeks of gestation for individual woman [15]. The computed GWG per week and the BMI calculated from the self-reported prepregnancy weight were used to classify women as having either excessive or normal/insufficient rate of weight gain using the Institute of Medicine 2009 GWG Guidelines [16].

#### Postnatal exposures

Offspring weight and length/height recorded at birth, 1, 2, 3, 4, and 4.5 years were used in the current analysis. Adiposity at 4.5 years was assessed using the sum of skinfold thicknesses measured at four sites (biceps, triceps, suprailiac, and subscapular) and BMI. Breastfeeding duration data were analyzed as a dichotomous variable: <3 versus ≥3 months of any (exclusive or partial) breastfeeding.

#### Infant genotype data

Umbilical cord DNA samples from the children were genotyped using the Illumina OmniExpress + exome array covering ~1 million SNPs. For quality control, SNPs, with call rates < 95%, or MAF < 5%, or those failing Hardy–Weinberg equilibrium were excluded. Principal components analysis was used to confirm self-reported race/ancestry. Alleles on the positive strand were reported as per the hg19 build of the human genome assembly. A total of 613,519 SNPs were available for analysis after union of SNPs that passed quality control checks, in each of the three racial groups.

### Outcomes

#### IMCL assessment by MRS

Prescan protocols for subject preparation are provided in the online supplementary section. All scans were performed without sedation. Following T1-weighted axial localization, point-resolved spectroscopy scans were performed on a 1 × 1 × 1 cm^3^ voxel placed in the soleus muscle and acquired with TR = 2000 ms, TE = 33 ms and N_acq_ = 24. Spectra were quantified using LC-Model [17]. T2 correction of the IMCL and water peaks was performed using T2 values reported in the literature [1]. The IMCL peak was normalized by the water peak from a water unsuppressed scan, and expressed as a percentage of the water signal from the same voxel.

#### Metabolic assessments

Fasting blood was collected using a Microvette 300FH collection tube at 6 years (*n* = 485). Glucose was measured from plasma using colorimetric assay on Beckman LX20 Pro analyzer (KKH) and Beckman AU5800 analyzer (NUH). Insulin was measured from serum stored at −80 °C using sandwich immunoassay on Beckman DXL800 analyzer (Beckman Coulter). Fasting glucose and insulin were used to calculate IR using the Homeostatic Model Assessment (HOMA): HOMA-IR = Fasting glucose (mmol/l) × Fasting insulin (mU/l)/22.5 [18].

### Statistical analysis

Statistical analyses were primarily performed using IBM SPSS Statistics for Windows, Version 23.0 (IBM Corp, Armonk, NY). Differences in maternal and offspring demographic and clinical characteristics among racial groups were tested using one-way ANOVA. Differences in the distribution of categorical variables across the racial groups were examined using χ^2^ tests.

#### Association of prenatal and postnatal factors with IMCL

General linear models were constructed to answer the following questions: (1) Which prenatal factors are associated with offspring IMCL? (2) Is postnatal breastfeeding duration associated with offspring IMCL? (3) Is rapid postnatal growth associated with offspring IMCL? and (4) Do these associations hold after adjusting for current BMI?

The prenatal exposures were collected at different times over the course of pregnancy. Demographic information and BMI were available at booking in the first trimester. The linear rate of gestational weight was calculated between 15 and 25 weeks. OGTT was performed in the 26th week of pregnancy. We constructed stepwise models (shown in Table 2) to understand how the estimates change when exposures collected at different time-points are added to the model, since they could potentially lie in the causal pathway between earlier exposures and offspring IMCL. The prenatal exposures included in the models were as follows: Model 1a: race, maternal education and maternal BMI; Model 1b: race, maternal education, maternal BMI and GWG rate; Model 1c: race, maternal education, maternal BMI, GWG rate, fasting glucose (26 weeks) and 2-h glucose (26 weeks). To evaluate if these factors were uniquely associated with offspring IMCL, independent of current BMI, we also ran an additional model (1d) that included current BMI as a covariate along with the exposures in model 1c.

**Table 2:** Associations of prenatal exposures with offspring IMCL (% of water) at 4.5 years

	Model 1a		Model 1b		Model 1c		Model 1d	
Exposures	*B* (95% CI)	*p*	*B* (95% CI)	*p*	*B* (95% CI)	*p*	B (95% CI)	*p*
Race								
Chinese vs Indian	−0.136 (−0.229, −0.043)	**0.004**	−0.111 (−0.202, −0.019)	**0.018**	−0.098 (−0.197, 0.001)	**0.053**	−0.103 (−0.200, −0.006)	**0.037**
Malay vs Indian	−0.156 (−0.255, −0.056)	**0.002**	−0.129 (−0.227, −0.031)	**0.010**	−0.107 (−0.218, 0.003)	**0.057**	−0.119 (−0.228, −0.010)	**0.032**
Education								
<12 years vs ≥12 years	0.018 (−0.052, 0.088)	0.614	0.000 (−0.070, 0.070)	0.994	0.004 (−0.070, 0.078)	0.915	0.012 (−0.06, 0.085)	0.736
Maternal BMI, kg/m^2^	0.007 (0.000, 0.015)	**0.045**	0.006 (−0.001, 0.013)	0.098	0.004 (−0.004, 0.012)	0.288	0.002 (−0.006, 0.010)	0.665
GWG rate, kg/week			0.396 (0.089, 0.702)	**0.012**	0.396 (0.069, 0.724)	**0.018**	0.319 (−0.007, 0.645)	**0.055**
OGTT (26 weeks)								
Fast glucose, mmol/l					0.032 (−0.046, 0.110)	0.419	0.027 (−0.049, 0.104)	0.483
2 h glucose mmol/l					0.003 (−0.025, 0.031)	0.822	0.001 (−0.027, 0.028)	0.964
Offspring BMI (4.5 years)							0.027 (0.009, 0.045)	**0.003**

The columns indicate unstandardized regression coefficients from stepwise general linear models. Each column corresponds to a different model. Blank rows indicate that the exposure is not used in the model. Bold values indicate statistical significance *P* < 0.05

The associations of postnatal factors with offspring IMCL are shown in Table 3. Model 2a shows the association of breastfeeding duration on offspring IMCL (adjusted for race). Model 2b additionally takes current BMI into account.

**Table 3:** Associations of postnatal exposures with offspring IMCL (% of water) at 4.5 years

	Model 2a		Model 2b	
Exposures	*B* (95% CI)	*p*	*B* (95% CI)	*p*
Race				
Chinese vs Indian	−0.137 (−0.223, −0.051)	**0.002**	−0.129 (−0.214, −0.045	**0.003**
Malay vs Indian	−0.153 (−0.245, −0.062)	**0.001**	−0.158 (−0.247, −0.068)	**0.001**
Breastfeeding duration				
<3 months vs ≥3 months	0.065 (0.001, 0.128)	**0.045**	0.042 (−0.021, 0.106)	0.188
Offspring BMI (4.5 years)			0.028 (0.012, 0.044)	**0.001**
	Model 3a		Model 3b	
Exposures	B (95% CI)		B (95% CI)	
Race				
Chinese vs Indian	−0.140 (−0.253, −0.026)	**0.017**	−0.145 (−0.259, −0.032)	**0.013**
Malay vs Indian	−0.236 (−0.357, −0.114)	<**0.001**	−0.237 (−0.358, −0.115)	**<0.001**
Sex				
Boys vs girls	0.002 (−0.079, 0.083)	0.964	−0.002 (−0.083, 0.079)	0.957
Maternal BMI (kg/m^2^)	0.007 (−0.002, 0.015)	0.134	0.005 (−0.003, 0.014)	0.229
GWG rate (kg/week)	0.225 (−0.180, 0.630)	0.274	0.176 (−0.003, 0.014)	0.397
Conditional relative weight gain				
0–1 years	0.029 (−0.010, 0.067)	0.144	0.002 (−0.051, 0.055)	0.952
1–2 years	0.032 (−0.006, 0.070)	0.096	0.008 (−0.042, 0.058)	0.755
2–3 years	0.052 (0.012, 0.093)	**0.012**	0.031 (−0.018, 0.080)	0.208
3–4 years	0.017 (−0.027, 0.060)	0.449	−0.003 (−0.053, 0.047)	0.142
Offspring BMI (4.5 years)			0.028 (−0.010, 0.066)	0.142

The columns indicate unstandardized regression coefficients from stepwise general linear models. Each column corresponds to a different model. Blank rows indicate that the exposure is not used in the model. Bold values indicate statistical significance *p* < 0.05

To assess the associations of rapid postnatal weight gain in early childhood on IMCL, conditional relative weight gain was evaluated for annualized intervals between birth and 4 years. Conditional relative weight gain at any time-point is defined as the standardized residual of the current weight regressed on the current height and all previous weight and height measurements [19]. It measures the deviation from the expected weight gain in an interval, given the child’s current height and previous growth measures. Thus, a high conditional weight gain in a particular interval indicates faster than expected growth, given the individual’s previous growth history. The advantage of conditional weight gain over a simple difference in weight in any specific time interval is that the former measure is completely uncorrelated to the previous growth history, and conditional scores computed in another time interval. Hence, they can be used to evaluate the independent associations of growth in different intervals within a single model. Model 3a shows the associations of conditional relative weight gain in annual intervals between 0 and 4 years with IMCL (adjusted for race, sex, maternal BMI, and GWG rate). Model 3b additionally takes current BMI into account.

#### Genome-wide association analysis (GWAS)

GWAS analysis was conducted on 252 subjects who had both imaging and genotype data. Linear regression of log-transformed IMCL was performed at each SNP, adjusting for race, maternal BMI, GWG, and sex. Since no SNP crossed the genome-wide cutoff (*p* < 8.1497e−08), (Supplementary Fig. S1), a generalized linear model with elastic net (GLMEN) regularization in MATLAB was derived using the top 100 SNPs (based on single SNP association *p*-values) to identify SNPs associated with IMCL [20]. Detailed modeling information can be found in the online supplementary section.

#### Associations of IMCL with metabolic outcomes at age 6

Associations of IMCL with metabolic outcomes were evaluated by linear regression, after adjusting for the hospital site.

## Results

Comparison of the demographic, maternal, and clinical characteristics of the children with IMCL data (*n* = 277), compared with children in the cohort from whom IMCL data were not available (*n* = 914), is shown in Supplementary Table S1. There were racial differences in the two categories with a lower proportion of Chinese (49.5% vs 58.8%) and a higher proportion of Malays (31.4% vs 23.5%) among children with IMCL data. Their mothers also had higher BMI at booking (24.4 ± 5.1 vs 23.4 ± 4.6 kg/m^2^, *p* = 0.003) and lower educational attainment (50.9% vs 39.9% mothers with <12 years of education, *p* = 0.001). There was also a lower proportion of primiparous mothers (39.5% vs 46.5%, *p* = 0.04) and boys (46.2% vs 54.6%, *p* = 0.014) among the group with IMCL data. However, there were no differences in offspring weight, height, BMI, or adiposity (sum of skinfolds) at 4.5 years between the two groups.

The complete maternal and offspring characteristics of the children with IMCL data, stratified by race, are shown in Table 1. Malay mothers were younger than both Chinese and Indian mothers. Chinese mothers had the lowest BMI. Indian mothers had higher fasting glucose than Malay and Chinese mothers. Both Indian and Chinese mothers had higher 2-h glucose compared with Malay mothers. There was a lower proportion of Chinese mothers and a higher proportion of Malay mothers with lower educational attainment, excessive rate of GWG and shorter duration of breastfeeding. The gestational age was higher in Chinese than in Malays. Indian children were taller than Malay and Chinese children at age 4.5, although there were no racial differences in weight or BMI.

The mean ± sd IMCL levels in the cohort was 0.481 ± 0.279% of water.

### Associations of prenatal factors and birth outcomes on IMCL accumulation at 4.5 years

The associations of prenatal factors on offspring IMCL at 4.5 years were examined (Table 2). Both race and maternal BMI at booking were independently associated with offspring IMCL levels. IMCL levels were higher in Indian children compared with Malay and Chinese children. Higher maternal BMI was linked to elevated offspring IMCL. The association of IMCL with maternal BMI was lost after including GWG rate (15–35 weeks) and maternal glycaemia (26th week) in the model. GWG rate was positively associated with IMCL. The associations of IMCL with race and GWG rate continued to hold after taking current offspring BMI into account.

### Postnatal factors and IMCL accumulation at 4.5 years

The associations of postnatal factors on offspring IMCL at 4.5 years are summarized in Table 3. A breastfeeding duration of <3 months was associated with higher IMCL levels. Faster weight gain in early childhood has been linked to increased risk of childhood obesity, as well as increased risk of cardiovascular disease risk in adulthood; hence we evaluated the association of IMCL with conditional relative weight gains assessed in annual intervals between birth and 4 years. Higher conditional relative weight gain from 2 to 3 years was significantly associated with higher IMCL levels at 4.5 years. The associations of offspring IMCL with both shorter breastfeeding duration and conditional elative weight gain between ages 2 and 3 were attenuated after adjusting for current offspring BMI. Offspring BMI was also associated with IMCL independent of race and breastfeeding duration.

### GWAS analyses of variability in IMCL accumulation in early childhood

**Fig. 1:**
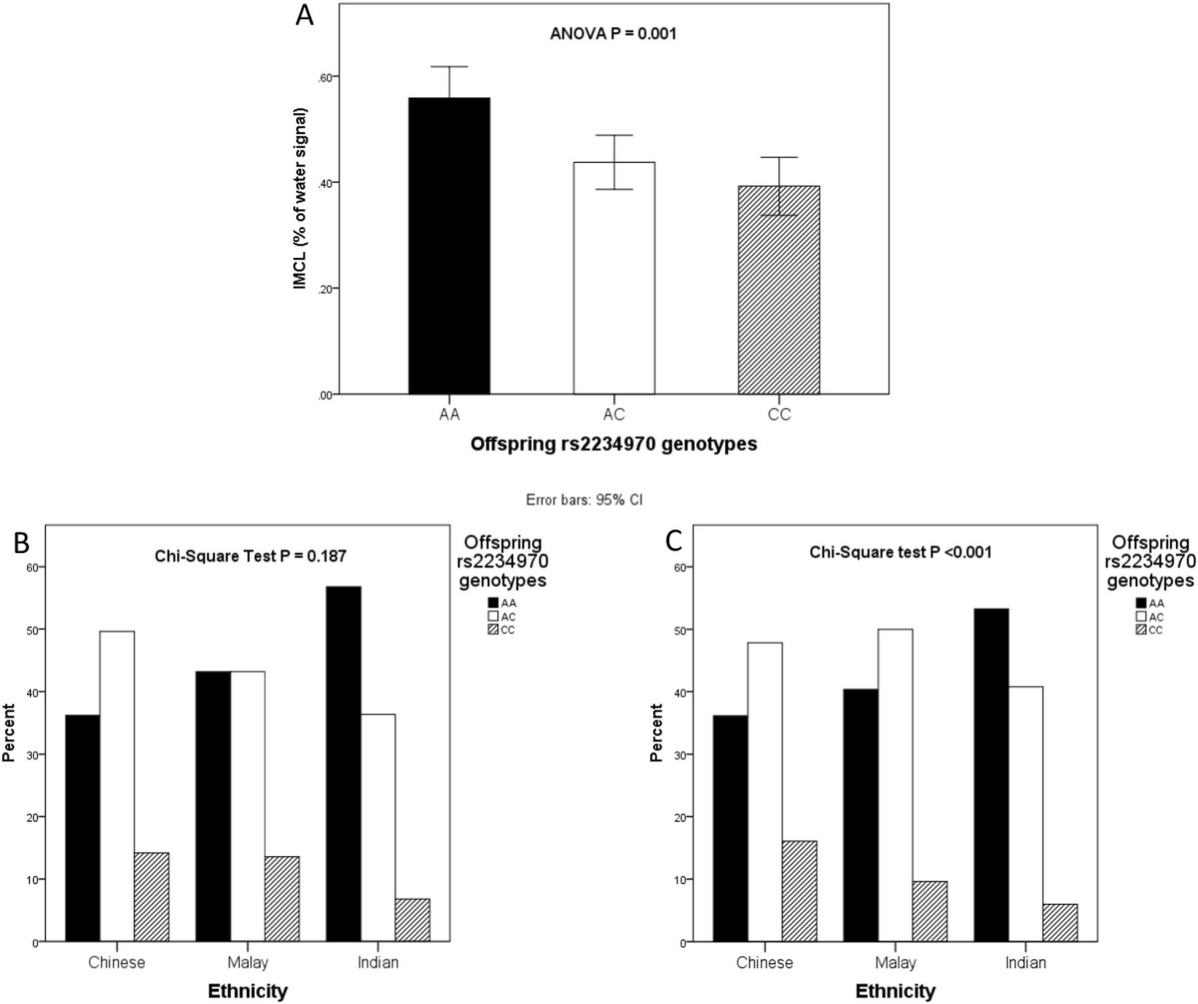
Association of rs2234970 SNP with IMCL and race. **a** Difference in offspring IMCL levels across offspring rs2234970 genotypes. **b** Racial differences in the distribution of the rs2234970 genotypes in the subset of children with both imaging and genotype date (*n* = 252). **c** Racial differences in the distribution of the rs2234970 genotypes in the entire GUSTO cohort (*n* = 1071)

No SNP crossed the genome-wide cutoff (*p* *<* 8.1497e−08), potentially due to the small number of subjects with both IMCL and genotype data (*n* = 252). A GLMEN regularization derived using the top 100 SNPs indicated 11 SNPs to be linked to variation in IMCL accumulation in early childhood (Supplementary Table S2). Of these, rs2234970 SNP (Met224Leu) was the only missense mutation. This SNP is located within the 5th exon of the Stearoyl Coenzyme Desaturase 1 gene (SCD1). SCD1 plays a key role in preventing lipotoxic effects as it converts saturated fatty acids to less harmful monounsaturated fatty acids, and has been previously linked to obesity and IR [21–23]. The mean and 95% CI of IMCL levels across rs2234970 genotypes are shown in Fig. 1a. An ANOVA test indicated a significant difference in means across the groups (*p* = 0.001). While IMCL was not normally distributed within all groups, the ANOVA test is robust to assumptions of normality. The data met the assumption of homogeneity of variance (Levene’s test *p* = 0.084). A post hoc test with Bonferroni correction showed children with homozygous major allele (AA) to have higher levels of IMCL than children with both AC (*p* < 0.004) and CC (*p* < 0.010) genotypes. Since only 252 offspring had both IMCL and genetic data, racial segregation was statistically difficult to conduct. Hence, we performed an extended analysis of the racial distribution of the rs2234970 genotypes in all subjects with genotype data in GUSTO cohort (*n* = 1071). The distribution of rs2234970 genotypes across the three racial groups is shown in Fig. 1b for subjects with IMCL data and in Fig. 1c for the entire cohort. A χ^2^ test indicated significant racial differences in the racial distribution of rs2234970 genotypes in the complete cohort (*p* < 0.001), but not in the subset with IMCL data (*p* = 0.187), probably due to the smaller sample size. In the entire cohort, Indian children carried two copies of the “A” risk allele at a higher frequency than Chinese (OR = 2.1, 95% CI (1.5–2.9)) or Malay (OR = 1.7, 95% CI (1.2–2.5)) children. We performed a mediation analysis, to evaluate the proportion of effect of Indian race mediated by the rs2234070 genotype. Bias corrected 95% confidence intervals of the indirect effects were obtained by bootstrapping with 1000 samples. The results of the analysis (complete results table shown in Supplementary Table S3), indicate rs2234970 genotype to mediate about 10.3% of the total effect of Indian race in IMCL at 4.5 years. We also tested for gene–environment interactions for all the prenatal and postnatal exposures, but found none of the interactions to be statistically significant.

### Associations of IMCL with metabolic outcomes

The IMCL levels at 4.5 years were not associated with fasting glucose, fasting insulin, or HOMA-IR assessed at age 6 (Table 4).

**Table 4:** Associations of offspring IMCL (% of water) at 4.5 years with metabolic outcomes assessed at age 6

Metabolic outcomes (6 years)	Model 4a		Model 4b		Model 4c	
	Fasting glucose (mmol/l)	*p*	Fasting insulin (mU/l)	*p*	HOMA-IR	*p*
*B* (95% CI) *p*-value	0.091 (−0.104, 0.287)	0.358	0.846 (−1.328, 3.020)	0.443	0.200 (−0.288, 0.688)	0.420

The columns indicate unstandardized regression coefficients indicating association of IMCL with different metabolic outcomes at age 6 (adjusted for hospital site)

## Discussion

Skeletal muscle IR plays a key role in the pathophysiology of type 2 diabetes [24], hence there is a strong interest to identify its determinants. IMCL has been implicated in the pathogenesis of skeletal muscle IR, and there is evidence suggesting that the increased metabolic risk associated with obesity [25], having first-degree relatives with type 2 diabetes [3], or having a personal history of gestational diabetes is partly conferred by increased IMCL accumulation [1]. However, the links between IMCL and IR are complex. High levels of IMCL can accumulate under conditions of elevated plasma free fatty acids and impaired fat oxidation, which is common in obese, sedentary states [26]. Paradoxically, high IMCL accumulation can also occur in endurance trained athletes who have enhanced fat oxidation [6]. IMCL levels are also higher in women, who have a higher capacity to mobilize the IMCL pool and oxidize fat during exercise, when compared with men [27]. In both the above cases, high IMCL does not adversely affect insulin sensitivity, suggesting that IMCL per se may just be a marker and may not be directly causing IR. The primary physiological role of IMCL is to serve as a buffer for plasma fatty acids before they are oxidized in the mitochondria [26]. Individuals who have high IMCL, but have reduced capacity to mobilize it under increased energy demands, may also have higher cytosolic accumulation of bioactive lipid intermediates of the esterification pathway like diacylglycerols and ceramides [28]. These lipid intermediates are known to impede insulin signaling and insulin-mediated glucose uptake by activating the protein kinase C system [28]. While IMCL has been extensively studied in adults and prepubertal children, there is very little knowledge of the determinants and metabolic consequences of IMCL accumulation in early childhood.

To the best of our knowledge, this study provides the first systematic study of IMCL levels in first 5 years of life and of potential underlying developmental, racial, and genetic determinants. The mean IMCL at 4.5 years was 0.481 ± 0.279% of water, which is about 40% of the degree of IMCL infiltration in healthy adult soleus muscle [1]. Our key finding is that racial differences in IMCL accumulation manifest in early life, with Indian children having higher IMCL levels than Chinese and Malay infants, recapitulating the phenotype observed in Asian adults [12]. Importantly, these racial differences persist even after adjusting for prenatal factors and the offspring’s current BMI. These results indicate that there may be racial differences in lipid availability, skeletal muscle substrate handling, and lipid buffering even during early childhood.

Among maternal factors, GWG rate was most strongly linked to offspring IMCL. The association was not significantly weakened after adjusting for current BMI. Among postnatal factors, shorter duration of breastfeeding and conditional relative weight gain between 2 and 3 years were associated with IMCL. However, these associations were lost after adjusting for current BMI, suggesting that these factors may be related to overall adiposity, which in turn influences IMCL accumulation. Offspring BMI was also associated with IMCL levels independent of race and breastfeeding duration.

While no SNP crossed genome-wide significance in the GWAS analysis, an elastic net regularization model with the top 100 SNPs identified an association between the missense SNP, rs2234970 in the *SCD1* gene and IMCL. To the best of our knowledge, there have been no previous reports linking rs2234970 to IMCL, hence potential mechanisms are necessarily speculative. The rs2234970 SNP has been found to be associated with SCD1 activity assessed by the SCD18 index in plasma phospholipids [29]. This index measures the SCD1 activity as the ratio of oleic acid (C18:1 n-9) to stearic acid (C18:0), which are the product and precursor of the SCD1 enzyme. The CC and AA genotypes were linked to highest and lowest SCD18 indices, respectively [29]. Muscle-specific SCD1 activity has been linked to increased lipid accumulation [30]. Hence, this is unlikely to explain our observation of the highest IMCL accumulation in the AA genotype and the lowest IMCL accumulation in the CC genotype. We believe that a more likely explanation lies in adipose tissue SCD1 activity. Lipid uptake and accumulation in the muscle in vivo is highly influenced by the plasma free fatty acid levels [31]. The rate of appearance of free fatty acids in the plasma is a function of the balance between lipolysis and reesterification in the adipose tissue [32]. Increased lipolysis and reduced reesterification have been observed in SCD1 knockout mice and after SCD1 inhibition in 3T3-L1 adipocytes [33]. Conversely, rosiglitazone treatment is known to increase SCD1 activity, reduce free fatty acid turnover, and improve peripheral insulin sensitivity, possibly by reducing fatty acid trafficking in the muscle [34, 35]. Some findings from the recent literature also indicate that type 2 diabetic subjects have lower SCD1 mRNA and protein expression in the subcutaneous adipose tissue [36]. Thus, increased plasma free fatty acid availability due to reduced reesterification and increased lipolysis in adipose tissue represents a potential mechanism through which lower SCD1 activity (as seen in the AA genotype) may increase muscle lipid accumulation.

We also found racial differences in the distribution of the rs2234970 genotype, with Indian children carrying two copies of the “A” risk allele (Fig. 1b, c) at a higher frequency than Chinese and Malay children. The rs2234970 genotype alone was found to mediate about 10.3% of the total effect of Indian race on IMCL levels at 4.5 years. This suggests that rs2234970 may represent just one of many SNPs or may be in linkage disequilibrium with other SNPs that regulate free fatty acid availability, and free fatty acid uptake, utilization and storage within the skeletal muscle in Indians.

While IMCL has been shown to be associated with IR in prepubertal childhood, our analysis indicated that IMCL in early childhood seems to be well tolerated. There were no associations between IMCL and fasting glucose, fasting insulin, and HOMA-IR measured at age 6. Further follow-up of these children at later time-points may provide more insights into whether IMCL tracks with age, and the levels at which it starts influencing insulin sensitivity.

The key strength of this study is the investigation of early determinants of IMCL accumulation including the in utero environment in a deeply phenotyped multiracial mother–offspring cohort. We found a range of developmental, racial, and genetic influences were associated with early childhood IMCL. Studying potentially pathogenic ectopic fat accumulation at a younger age might reflect better on early determinants than doing so later in the life course when the phenotype become confounded with other manifestations. The racial differences, in particular, have been subject to much study in understanding metabolic risk. In part, the differences appear to depend on different patterns of adiposity with Indians reported to be more likely to experience ectopic fat accumulation due to adipose tissue overflow [37]; observations in the current study supported these findings.

This study also has a few limitations. This was a secondary analysis, and the GUSTO study was not originally designed to answer these specific hypotheses. There were only 252 subjects with both imaging and genetic data, which limited the sample size for genetic investigations. Usable IMCL data were successfully collected from a small subset of the GUSTO cohort, hence children with and without IMCL data differed in their maternal characteristics, which may have biased the study outcomes. We have evaluated the associations of IMCL with a number of maternal and early life predictors, which may have increased the possibility of false positives.

In conclusion, we report that IMCL, a fat depot linked to increased metabolic risk in adults and preadolescents, can be quantified in early childhood using MRS. Indians accumulate IMCL at a higher level even in early childhood recapitulating adult phenotypes. This is partly due to Indians having increased odds of having one or more “A” risk allele of the rs2234970 genotype, which was linked to elevated IMCL. The associations of race and GWG rate on IMCL exist even after taking current BMI into account. Importantly, we also show that IMCL in early childhood seem to be fairly well tolerated and showed no associations with IR at age 6. These observations may partially explain the racial diversity in IMCL accumulation, and may offer new opportunities in screening children with an early tendency for accumulating fat in pathogenic depots.

## Supplementary information

Supplementary Materials
