# Supplementary material for: Determinants of intramyocellular lipid accumulation in early childhood
Source: Int J Obes (Lond). 2019 Aug 28;44(5):1141–51. doi: 10.1038/s41366-019-0435-8 (PMC7188663; doi:10.1038/s41366-019-0435-8)
Supplement: Supplementary file 1 — Supplementary Materials [file 41366_2019_435_MOESM1_ESM.docx]

**Online-only Supplementary Materials**

1. **Pre-scan Procedures: MR Spectroscopy of IMCL**

Investigating IMCL levels in preschool children is difficult, owing to the practical challenges of ensuring subject immobility without sedation for magnetic resonance imaging (MRI). To improve compliance of the children with the scan protocols, they were familiarized with MRI scan noise and procedures through training videos, a coloring book and also allowed to interact with a toy MRI scanner. All scans were performed without sedation on a Siemens Magnetom Skyra 3T scanner. They were accompanied by their parent for the entire duration of the scan. The children were placed head-first supine in the magnet. Imaging was performed using a 4-channel flex coil, wrapped around the left calf. After wrapping the coil, the foot was placed in a neutral position and the left lower leg immobilized with sandbags. A periscopic double mirror was placed on the head coil, which allowed the subject to view an animated movie on the projection screen for the duration the scan. This was found to significantly reduce subject motion within the scanner and increased subject cooperation.

1. **Genome-wide association analysis (GWAS) using generalized linear model with elastic net regularization (GLMEN)**

A generalized linear model with elastic net regularization (GLMEN) in MATLAB was derived using the top 100 SNPs (based on single SNP association p-values) to identify SNPs associated with IMCL^25^. We excluded the children with missing values of maternal ppBMI and GWG (gestational weight gain) and also with missing values of top 100 SNPs thereby leaving 222 subjects for subsequent analysis. The data was randomly partitioned into 80% (training) and 20% (test) subjects. Post-hoc analysis estimated the 95% confidence interval of elastic net coefficient, β, corresponding to each SNP from bootstrap resampling (10,000 datasets). We also assessed the effect of adding SNPs of lower significance (error SNPs) to the initial pool of SNPs for deriving Elastic Net Model. The SNPs with confidence intervals containing zero or showing change in β direction when error SNPs were added were dropped from the model. Finally, we validated the above model using test data which was not used in model training, and found a statistically significant adjusted coefficient of determination of 0.26 (p = 0.0003) between the estimated and actual IMCL values.

**
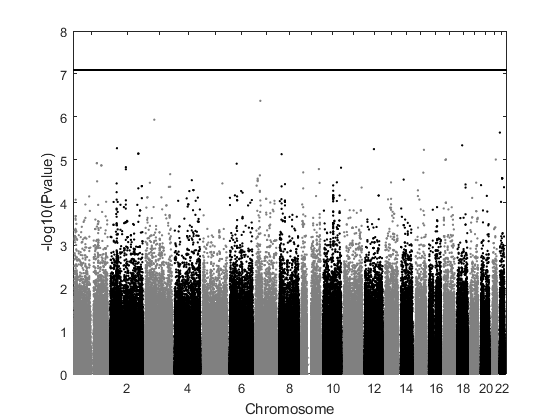
**

**Figure S1** Manhattan plot corresponding to single SNP association with log(IMCL). The -log10(p-value) corresponding to Regression Coefficient of SNP in Generalized Linear Model (log(IMCL)~SNP+confounders) is plotted against chromosomal position. The Bonferroni threshold (8.1497e-08) is represented by the horizontal line.

**Table S1.** Differences in the maternal and offspring characteristics between children in the GUSTO cohort from whom IMCL data was available compared to children from whom IMCL data was not available.

| **Characteristics** | **IMCL data not available (4.5y)** | **IMCL data available**  **(4.5y)** | **P-value** |
| --- | --- | --- | --- |
| **N** | 914 | 277 |  |
| **Race**  - Chinese  - Malay  - Indian | 58.8%  23.5%  17.6% | 49.5%  31.4%  19.1% | **0.029** |
| **Maternal Age (years)** | 30.8 (5.0) | 30.6(5.4) | 0.609 |
| **Maternal BMI (kg/m^2^)** | 23.4 (4.6) | 24.4 (5.1) | **0.003** |
| **Maternal Education <12y** | 39.9% | 50.9% | **0.001** |
| **Maternal Fasting Glucose (mmol/l)** | 4.4 (0.5) | 4.4 (0.5) | 0.859 |
| **Maternal 2h Glucose (mmol/l)** | 6.6 (1.5) | 6.4 (1.4) | 0.054 |
| **GWG rate (kg/week)** | 0.475 (0.115) | 0.462 (0.109) | 0.134 |
| **Excessive GWG rate (IOM 2009)** | 47.4% | 47.6% | 0.952 |
| **Primiparity** | 46.5% | 39.5% | **0.040** |
| **Gestational Age (days)** | 270.5 (11.8) | 269.5 (12.2) | 0.220 |
| **Male Sex** | 54.6% | 46.2% | **0.014** |
| **Birth Weight (g)** | 3067 (487) | 3048 (483) | 0.570 |
| **Breast-feeding Duration < 3 months** | 43.3% | 49.2% | 0.101 |
| **Weight, 4.5 y (kg)** | 17.3 (3.0) | 17.6 (3.1) | 0.213 |
| **Height, 4.5 y (cm)** | 105.5 (4.5) | 105.5 (4.3) | 0.951 |
| **BMI, 4.5 y (kg/m^2^)** | 15.5 (1.9) | 15.7 (2.0) | 0.086 |
| **Sum-of-skinfolds, 4.5 y (mm)** | 30.6 (10.1) | 31.6 (12.2) | 0.191 |

| **SNP** | **Chrmo-some** | **Chromo-some_position** | **Strand** | **Sequence** | **Overlapping Features** | **SNP function** | **Gene description** | **p-value** | **beta** | **CI95%** | **CI95%** |
| --- | --- | --- | --- | --- | --- | --- | --- | --- | --- | --- | --- |
| rs5749060 | 22 | 17846171 | - | AG | intron of CECR2 | - | Cat Eye syndrome Chromosome Region, candidate 2 | 2.34E-06 | 0.032659 | 0.012142 | 0.066414 |
| rs12004434 | 9 | 129058924 | - | AG | Intergenic region | - | - | 1.66E-05 | -0.02619 | -0.05993 | -0.00142 |
| rs4903608 | 14 | 40784744 | + | AG | Intergenic region | - | - | 2.92E-05 | -0.0448 | -0.10804 | -0.00747 |
| rs2234970 | 10 | 102116311 | + | AC | Exon within SCD | Missense | Stearoyl-CoA Desaturase (delta-9-desaturase) | 3.38E-05 | -0.02535 | -0.06044 | -0.00227 |
| rs871683 | 3 | 32832864 | + | AG | Intergenic region | - | - | 3.46E-05 | -0.0396 | -0.08508 | -0.01072 |
| rs11993621 | 8 | 42238574 | - | AG | region is 3899 bp upstream of DKK4 | - | Dickkopf WNT Signaling Pathway Inhibitor 4 | 3.73E-05 | -0.02727 | -0.07854 | -0.00204 |
| rs4391 | 22 | 48653779 | + | AC | Intergenic region | - | - | 4.4E-05 | -0.05459 | -0.10488 | -0.00978 |
| rs10224342 | 7 | 33869805 | - | AG | Intergenic region | - | - | 5.32E-05 | -0.03871 | -0.08835 | -0.01536 |
| rs17036385 | 2 | 47461253 | - | AC | intron of LOC101927043 | - | Uncharacterized LOC101927043 | 5.75E-05 | -0.06542 | -0.11652 | -0.02273 |
| rs1689549 | 3 | 14837473 | - | AG | Intergenic region | - | - | 7.57E-05 | 0.024042 | 0.00238 | 0.06061 |
| rs7651619 | 3 | 118667405 | + | AG | intron of IGSF11 | - | Immunoglobulin Superfamily Member 11 | 7.58E-05 | 0.044557 | 0.017676 | 0.097528 |

**Table S2.** Summary of chromosomal positions, gene annotations, SNP function, p-value of single SNP association with log10(IMCL), and regression coefficients along with 95% confidence interval of significant SNP associated with log10(IMCL) derived from Generalized Linear Model with Elastic Net Regularization.

**Table S3.** Direct and Indirect effects of Indian race on offspring IMCL (% of water) at 4.5 years mediated by the rs2234970 SNP

| Effect | B | 95% CI | |
| --- | --- | --- | --- |
| Direct Effect | 0.174 | 0.085 | 0.264 |
| Indirect Effect | 0.020 | 0.004 | 0.044 |
| Total Effect | 0.194 | 0.104 | 0.285 |
| Ratio of Indirect to Total Effect | 0.103 | 0.016 | 0.393 |
